# *Notes from the Field:* Emergency Department Use During the Los Angeles County Wildfires, January 2025

**DOI:** 10.15585/mmwr.mm7403a2

**Published:** 2025-02-06

**Authors:** Emily Kajita, Karen Chang, Vannalyn de Leon, Wesley Moss, Michael Lim, Sharon Balter, Annabelle de St. Maurice

**Affiliations:** ^1^Los Angeles County Department of Public Health, Los Angeles, California; ^2^Career Epidemiology Field Officer Program, CDC.

**Summary**

**What is already known about this topic?**

Syndromic surveillance provides timely information about the health impacts associated with the occurrence of natural disasters.

**What is added by this report?**

Immediately after the 2025 Los Angeles County wildfires began, all-cause emergency department (ED) encounters decreased by 9%, concomitant with an eightfold increase in the average percentage of ED encounters classified as wildfire-associated. During the analysis period, no differences were observed in the average percentage of ED encounters for cardiorespiratory illnesses.

**What are the implications for public health practice?**

Jurisdictions can use syndromic surveillance in real time to estimate effects of wildfires on health care use to identify opportunities for intervention, such as sharing communications with the general public about the importance of minimizing exposures to wildfire smoke, especially during the first few days of wildfire events.

On January 7, 2025, wildfires erupted in the Pacific Palisades and in Eaton Canyon in Los Angeles County (LAC), California. Fueled by dry weather conditions and Santa Ana winds with speeds of 60–80 mph (97–129 kph) and gusts up to 100 mph (161 kph), the fires burned approximately 40,000 acres, destroyed approximately 16,000 structures, and killed at least 29 persons (*1*). Near real-time surveillance of health outcomes during and after wildfires can estimate effects on health care use, serve as an early warning for acute health impacts, and identify opportunities for intervention.

## Investigation and Outcomes

### Data Sources and Analysis

The LAC Department of Public Health’s Syndromic Surveillance program receives and analyzes data from 90% of emergency departments (EDs) in LAC, representing 94% of all LAC ED encounters. For this study, syndromic surveillance data were reviewed to examine trends in all-cause and wildfire-associated ED encounters contemporaneous with the LAC wildfires. Encounters were classified as wildfire-associated if fire or smoke inhalation–related terms (*2*) were present in chief complaints and diagnoses.* Three periods were analyzed: December 17, 2024–January 6, 2025 (baseline: a 3-week period before the wildfires began); January 7, 2025–January 12, 2025 (phase 1: the first 6 days of the wildfires); and January 13, 2025–January 19, 2025 (phase 2: the following 7 days). These periods were designated retrospectively and selected based on the largest changes in all-cause and wildfire-associated ED encounters. The average number of all-cause ED encounters and the average percentage of ED encounters that were wildfire-associated were calculated for each period. Analyses of burn-, eye-, cardiovascular-, and respiratory-related ED encounters were conducted using local queries adapted from cross-jurisdictional collaborations (*3*). Air quality index (AQI) data were obtained from one Environmental Protection Agency (EPA) air quality monitoring station located in downtown Los Angeles.^†^This study was reviewed by the Los Angeles County Department of Public Health, and was deemed nonresearch public health surveillance and exempt from Human Research Protection Office review.^§^

### Changes in AQI and Distributions of All-Cause and Wildfire-Associated ED Encounters

The average number of daily all-cause ED encounters decreased an absolute 9% from baseline to phase 1 (representing 91% of baseline encounters), and then increased to 95% of baseline encounters in phase 2. The average percentage of ED encounters that were wildfire-associated increased eightfold, from 0.06% at baseline to 0.52% in phase 1, and then decreased but remained elevated at 0.20% in phase 2. The percentage of wildfire-associated ED encounters peaked at 1.01% on January 8, 2025 (Figure). The increase in average daily AQI aligned with the increase in average percentage of wildfire-associated ED visits: average daily AQI increased from 75 (moderate AQI level of concern) at baseline to 110 (unhealthy for sensitive groups) during phase 1 and returned to moderate (58) in phase 2.

**FIGURE:**
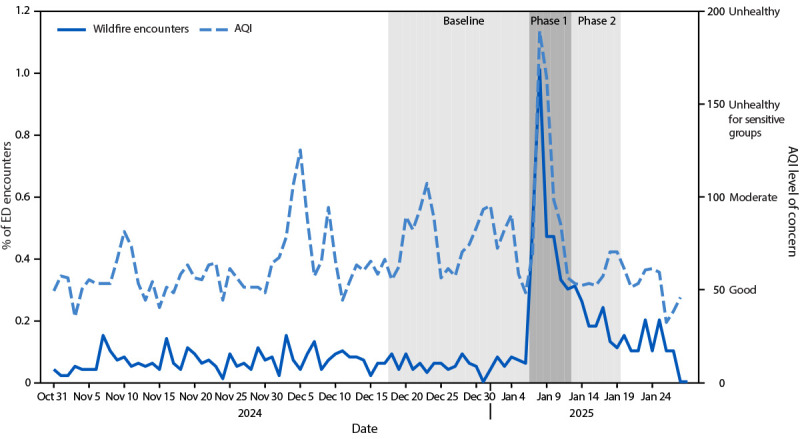
Daily percentage of emergency department encounters that were wildfire-associated and air quality index values* during three periods related to wildfires^†^ — Los Angeles County, California, October 31, 2024–January 28, 2025 **Abbreviations**: AQI = air quality index; ED = emergency department. * Unitless AQI levels of concern depicted are defined as follows: good = 0–50; moderate = 51–100; unhealthy for sensitive groups = 101–150; unhealthy = 151–200; very unhealthy = 201–300; and hazardous = ≥301. ^†^ Baseline period = 3 weeks before the onset of the fires (December 17, 2024–January 6, 2025); phase 1 = first 6 days of the fires (January 7–12, 2025); and phase 2 = 7 days after phase 1 (January 13–19, 2025).

### Types of ED Encounters and Injuries

Small differences were noted among baseline, phase 1, and phase 2 in the average percentages of burn-related injuries (0.97%, 1.30%, and 1.04%, respectively) and eye-related ED encounters (1.09%, 1.46%, and 1.17%, respectively). No patterns of increase were noted in cardiovascular or asthma and other respiratory subcategories such as respiratory distress, acute bronchitis, shortness of breath, cough, or sore throat.

## Preliminary Conclusions and Actions

Although the overall percentage of wildfire-associated ED encounters increased with the onset of the LAC fires, all-cause ED encounters initially decreased. These findings align with studies demonstrating similar decreases in ED encounters immediately after natural disasters, including heavy smoke events due to wildfires (*4*). The observed decrease could be due to evacuations leading to displacements, alterations in activity patterns (e.g., school and business closures) as well as increased avoidance of or challenges accessing health care or EDs. LAC residents might have sought care in clinics, urgent care centers, or EDs in neighboring counties as an alternative to visiting LAC EDs, and those encounters would not be recorded in these data.

Although increases in ED encounters related to asthma and other cardiorespiratory subcategories have been reported by other local health jurisdictions after wildfire events even a considerable distance away (*5*), LAC data did not demonstrate similar increases. Small differences might not be detectable for wildfires occurring during peak viral respiratory activity, and smoke distribution might be affected by the unique topography of the Los Angeles basin and recurring Santa Ana winds. Small numbers of encounters might also be attributed to inconsistent coding by clinicians of terms related to smoke or fire exposures.

In light of these limitations, the data likely underestimate the prevalence of wildfire-associated ED encounters. These data demonstrate that the wildfires were associated with a decrease in total ED encounters across LAC, and that wildfire-associated ED encounters were temporally associated with worsening air quality. Further analyses are planned to identify which illnesses have most affected specific populations. Additional data elements could be incorporated to further characterize short- and long-term health consequences. In anticipating wildfires and preparing for responses, developing timely communications about wildfire smoke, including risk for exposure and precautions could mitigate risk.

## Footnotes

* The LAC adaptation of the “fire and smoke inhalation V1” Council of State and Territorial Epidemiologists’ query includes additional exclusion terms to counter misclassifications due to unrelated references to fire and smoke.

† AQI is calculated using the concentrations of pollutants in parts per million (*μ*g/m^3^); for this report, unitless AQI levels of concern were categorized and defined as follows: 0–50 = good; 51–100 = moderate; 101–150 = unhealthy for sensitive groups; 151–200 = unhealthy; 201–300 = very unhealthy; and ≥301 = hazardous. Data are available from EPA. AirNow, an Internet database, is available at https://airnowapi.org/ (Accessed February 26, 2023). Data from the selected monitor (Los Angeles – North Main Street, AQI ID: 060371103) represent a surrogate for trends and do not reflect peak measurements observed in all wildfire-impacted areas. EPA monitors do not measure all toxic pollutants released in urban wildfires and would have been subject to planned and unplanned power outages occurring in wildfire adjacent communities.

§ 45 C.F.R part 46.102(l)(2).
